# Dosimetric comparison of five different radiotherapy treatment planning approaches for locally advanced non‐small cell lung cancer with sequential plan changes

**DOI:** 10.1111/1759-7714.15137

**Published:** 2023-10-16

**Authors:** Masahide Saito, Takafumi Komiyama, Kan Marino, Shinichi Aoki, Tomoko Akita, Masaki Matsuda, Naoki Sano, Hidekazu Suzuki, Ueda Koji, Hikaru Nemoto, Hiroshi Onishi

**Affiliations:** ^1^ Department of Radiology University of Yamanashi Yamanashi Japan

**Keywords:** 3DCRT, IMRT, lung cancer, radiotherapy, VMAT

## Abstract

**Background:**

The purpose of this study was to compare the dosimetric characteristics of five different treatment planning techniques for locally advanced non‐small cell lung cancer (LA‐NSCLC) with sequential plan changes.

**Methods:**

A total of 13 stage III NSCLC patients were enrolled in this study. These patients had both computed tomography (CT) images for initial and boost treatment plans. The latter CT images were taken if tumor shrinkage was observed after 2 weeks of treatment. The prescription dose was 60 Gy/30 Fr (initial: 40 Gy/20 Fr, and boost: 20 Gy/10 Fr). Five techniques (forward‐planed 3‐dimensional conformal radiotherapy [F‐3DCRT] on both CT images, inverse‐planned 3DCRT [I‐3DCRT] on both CT images, volumetric modulated arc therapy [VMAT] on both CT images, F‐3DCRT on initial CT plus VMAT on boost CT [bVMAT], and hybrid of fixed intensity‐modulated radiotherapy [IMRT] beams and VMAT beams on both CT images [hybrid]) were recalculated for all patients. The accumulated doses between initial and boost plans were compared among all treatment techniques.

**Results:**

The conformity indexes (CI) of the planning target volume (PTV) of the five planning techniques were 0.34 ± 0.10, 0.57 ± 0.10, 0.86 ± 0.08, 0.61 ± 0.12, and 0.83 ± 0.11 for F‐3DCRT, I‐3DCRT, VMAT, bVMAT, and hybrid, respectively. In the same manner, lung volumes receiving >20 Gy (V_20Gy_) were 21.05 ± 10.56%, 20.86 ± 6.45, 19.50 ± 7.38%, 19.98 ± 10.04%, and 17.74 ± 7.86%. There was significant improvement about CI and V_20Gy_ for hybrid compared with F‐3DCRT (*p* < 0.05).

**Conclusion:**

The IMRT/VMAT hybrid technique for LA‐NSCLC patients improved target CI and reduced lung doses. Furthermore, if IMRT was not available initially, starting with 3DCRT might be beneficial as demonstrated in the bVMAT procedure of this study.

## Graphical abstract

The study examined five planning techniques for advanced lung cancer, evaluating dosimetric traits with plan changes and extensions. It found plan changes useful for target alterations, but a single re‐planning in offline‐adaptive radiotherapy might not minimize lung dose adequately. The IMRT/VMAT hybrid showed enhanced conformity and lower lung doses, and starting with 3DCRT is recommended if IMRT is not available initially.

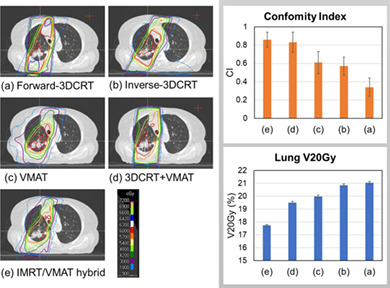

## INTRODUCTION

The importance of chemoradiotherapy for the treatment of locally advanced non‐small cell lung cancer (LA‐NSCLC) has increased since the PACIFIC trial demonstrated an improved prognosis.^1^ In order to implement this treatment regimen, it is important to prevent radiation pneumonitis during radiotherapy. In general, the occurrence of radiation pneumonitis has been suggested to correlate with normal lung dose indices.^2^, ^3^ In particular, numerous reports on V_20Gy,_ ^2^ mean lung dose (MLD), and V_30Gy_ have revealed an association with the incidence of radiation pneumonitis.^4^ Therefore, in treatment planning during radiotherapy against LA‐NSCLC, it is important to reduce the normal lung dose to the maximum feasible extent while administering the necessary dose to the target.

Furthermore, during radiotherapy treatment planning for LA‐NSCLC, the cardiac dose must also be closely monitored, as the RTOG0617 study suggested that the cardiac dose may contribute to overall survival.^5^, ^6^ The latest cardio‐oncology guidelines define a mean heart dose (MHD) of ≥25 Gy as very high risk, <25 Gy but >15 Gy as high risk, <15 Gy but >5 Gy as moderate risk, and <5 Gy as low risk.^7^ A literature review conducted from 2013 to 2020 reported an average cardiac dose of 10.3 Gy.^8^ Although there are cases in which cardiac dose reduction is difficult owing to the anatomical location of the lesion, it is important to reduce the cardiac dose to the maximum feasible extent.

Thus, there are various organs at risk (OARs) for radiotherapy in LA‐NSCLC, and various irradiation methods have been developed to mitigate the OARs. For example, intensity‐modulated radiotherapy (IMRT),^9^ volumetric modulated arc therapy (VMAT),^10^ the IMRT/VMAT hybrid technique,^11^ and three‐dimensional conformal radiotherapy (3DCRT) with an inverse plan (I‐3DCRT)^12^ have become available. However, only a few comprehensive studies have compared the dosimetric characteristics of these modern treatment plans. Furthermore, in actual clinical practice, treatment plans may need to be changed during the course of treatment because of the lack of manpower at the facility (i.e., starting with simple 3DCRT and changing to IMRT later) or the patient's anatomical changes (i.e., offline adaptive radiotherapy [ART]).^13^ To date, many planning studies have only evaluated single planning computed tomography (CT) images. However, they did not consider the doses to normal organs when sequential plan changes occurred, such as in the two‐step method. In particular, the dosimetric impact of switching from 3DCRT to VMAT during the treatment period has not been fully considered or compared with that of other techniques.

Therefore, the purpose of this study was to compare the dosimetric characteristics of five different treatment planning techniques for LA‐NSCLC with sequential plan changes.

## METHODS

### Study design

This study was approved by the Institutional Review Board (IRB) of the University of Yamanashi (no.: 2271). A total of 13 patients who underwent CRT for LA‐NSCLC between January 2016 and January 2020 at our hospital were included in the study, with different CT scans for the initial plan (pre 40 Gy) and boost plan (post 40 Gy). CT images were obtained using the Abches respiratory management device (APEX Medical, Tokyo, Japan).^14^ All patients were treated with involved‐field radiotherapy using inspiration breath‐hold technique. Therefore, all the CT images were acquired during the maximum inspiration phase for each patient.

### Treatment planning

**FIGURE 1:**
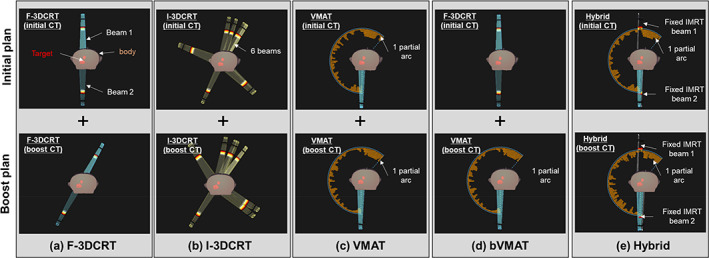
Representative arrangement for five treatment planning technique. The upper raw shows the treatment plan of the initial plan (40 Gy/20 Fr) and the lower raw shows the treatment plan of the boost plan (20 Gy/10 Fr). From left to right: (a) F‐3DCRT, (b) I‐3DCRT, (c) VMAT, (d) bVMAT, and (3) hybrid beam arrangement, are shown.

Treatment plans were recalculated for this study. We employed five different treatment planning methods (Figure 1). For all these methods, the prescribed dose was set at 60 Gy delivered over 30 fractions, with a planning target volume (PTV) D50% prescription. The PTV was determined by expanding the internal target volume (ITV) by 5 mm in all directions. In this study, all treatment planning CT images were obtained with inhalation breath‐hold for respiratory motion management. As a result, the ITV was smaller compared to irradiation under free breathing.^14^ The ITV was determined for each patient by adding any uncertainties related to breath‐holding (approximately 2–3 mm) from three consecutive CT images to the clinical target volume (CTV). Notably, CTVs were distinctly defined for primary tumors and lymph node metastases.

After the initial (40 Gy/20 Fr) and boost (20 Gy/10 Fr) plans were created for each CT image, deformable dose accumulation was performed on the boost CT image to create a summed dose distribution. Synergy with an agility gantry head (Elekta AB) was used for radiation therapy and the RayStation version 10A (RaySearch Laboratories) was used for the treatment planning. The X‐ray energy was 6 MV, the dose calculation algorithm was a collapsed cone convolution, and the grid size was 2 mm. Hybrid intensity‐ and structure‐based deformable image registration were used for deformable dose accumulation.

The five treatment planning methods follow.

#### Forward planned 3DCRT (F‐3DCRT)

The initial plan was formulated using two anterior–posterior (AP) irradiation directions, whereas the boost plan utilized two to four irradiation directions with an added oblique entrance to spare the spinal cord. A representative beam arrangement is illustrated in Figure 1a.

#### Inverse planned 3DCRT (I‐3DCRT)

In this method, beam alignment was performed using the 3DCRT optimization function in RayStation. We set six beams at 60‐degree intervals around the gantry to optimize the gantry angle, collimator angle, irradiation field shape, and monitor unit (MU) values. Because noncoplanar beams were not utilized, the couch angle remained fixed. The initial and boost plans were implemented using the same methodology. A representative beam arrangement can be seen in Figure 1b.

#### VMAT

1–2 partial arc VMAT was performed. The same geometry was applied to both the initial and boost plans. A representative beam arrangement is shown in Figure 1c.

#### F‐3DCRT + VMAT (boost VMAT: bVMAT)

For the initial plan, the F‐3DCRT with AP irradiation created in (a) was used. For the boost plan, the VMAT created in (c) was used. A typical beam arrangement is shown in Figure 1d.

#### IMRT/VMAT hybrid (hybrid)

The beams were configured using two fixed IMRT (AP irradiation) combined with 1–2 partial arc VMAT. The same geometry was maintained for both the initial and boosted plans. These plans were crafted using the co‐optimization function of RayStation. A representative beam arrangement is shown in Figure 1e.

### Dosimetric evaluation

In this study, dosimetric comparison was performed by summing the dose distributions of the initial and boost doses. For each dose, the PTV (D_98%_, D_2%_, D_mean_, and conformity index [CI]), lung (mean lung dose [MLD], V_20Gy_, and V_5Gy_), spinal cord D_max_, heart D_mean_, and esophageal D_mean_ were calculated. CI was calculated using the following formula:

$$\mathrm{CI}=\frac{{\mathrm{TV}}_{\mathrm{RI}}}{\mathrm{TV}}\times \frac{{\mathrm{TV}}_{\mathrm{RI}}}{V_{\mathrm{RI}}}$$

where, ${\mathrm{TV}}_{\mathrm{RI}}$ was the target volume covered by the reference isodose, *𝑇𝑉* was the target volume, and $V_{\mathrm{RI}}$ was the volume of the reference isodose. The reference isodose was 40 Gy for initial plan and 20 Gy for boost plan. In addition, total MU values were investigated to assess the complexity of treatment planning and the impact of low‐dose exposure. All statistical analyses were performed using the JMP Pro 17.0 (SAS institute) with a Wilcoxon text and a *p*‐value <0.05 was considered statistically different.

## RESULTS

Patient characteristics are shown in Table 1. Median age of the participants was 60 years (range: 48–60 years). The cohort comprised nine males and four females. Six patients were diagnosed with LA‐NSCLC stage IIIA and seven with stage IIIB. The lesion was located in the upper lobe in five, middle lobe in four, and lower lobe in four patients. CTV volume was 128.47 ± 200.78 cc for the initial and 69.50 ± 88.13 cc for boost plans (*p* < 0.05). PTV volume was 397.65 ± 384.51 cc for the initial and 287.29 ± 238.96 cc for boost plans (*p* < 0.05). Normal lung volume was 3889.07 ± 620.43 cc for the initial and 3882.59 ± 647.36 cc for boost plans (*p* = 0.946).

**TABLE 1:** Patient characteristics.

*n*	13
Age (median [range])	60 [48–80]
Sex	Male: 9, Female: 4
Stage	IIIA: 6, IIIB: 7
Tumor site	Upper lobe: 5 Middle lobe: 4 Lower lobe: 4
Clinical target volume (CTV) (mean ± s.d., [cc])	Initial: 128.47 ± 200.78 Boost: 69.50 ± 88.13 (*p* < 0.05)
Planning target volume (PTV) (mean ± s.d., [cc])	Initial: 397.65 ± 384.51 Boost: 287.29 ± 238.96 (*p* < 0.05)
Normal lung volume (mean ± s.d., [cc])	Initial: 3889.07 ± 620.43 Boost: 3882.59 ± 647.36 (*p* = 0.946)

**FIGURE 2:**
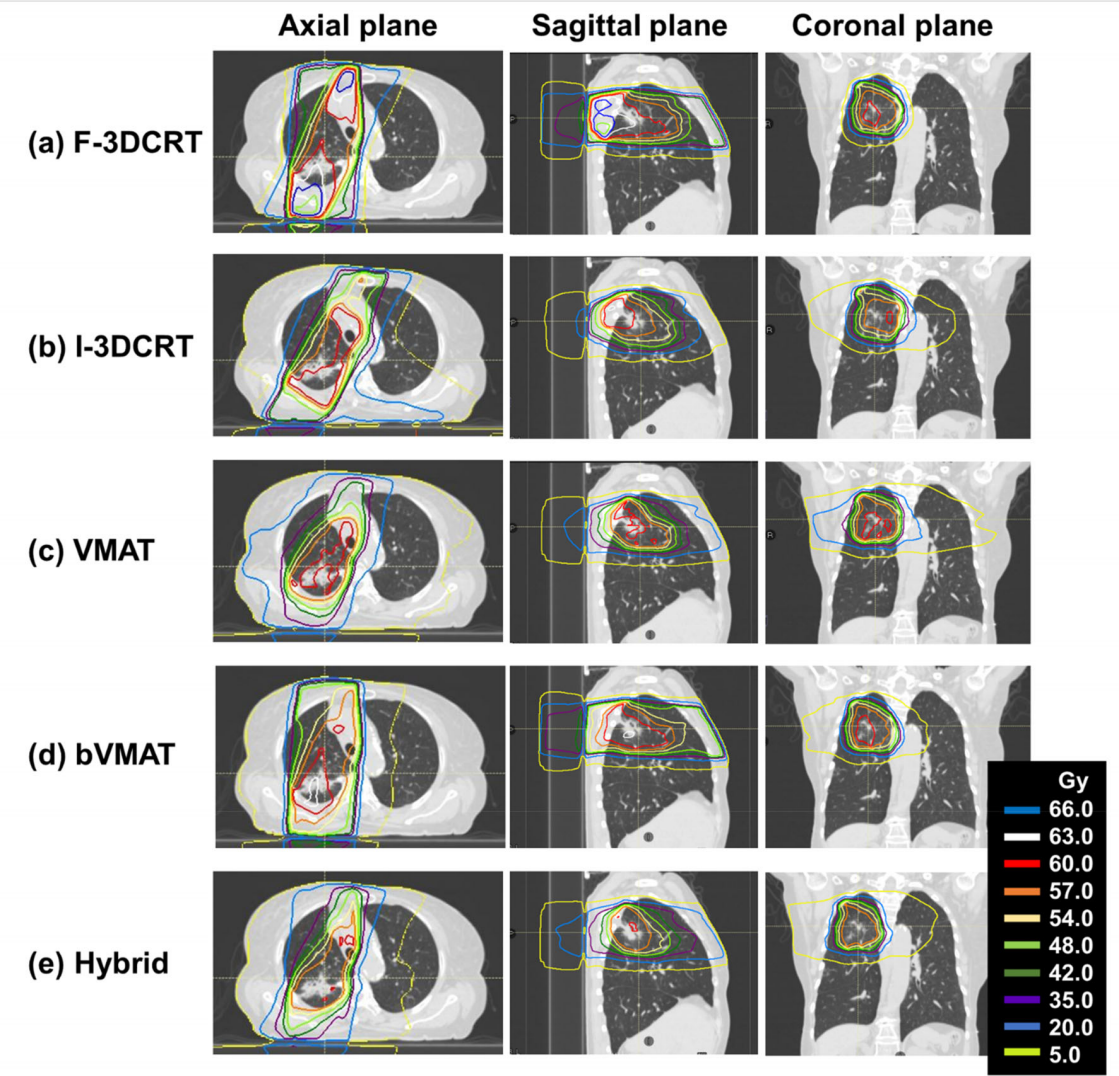
Representative dose distributions for each technique. From left to right, axial, sagittal and coronal planes are shown. From top to bottom, (a) F‐3DCRT, (b) I‐3DCRT, (c) VMAT, (d) bVMAT, and (e) hybrid results, showing the summation dose distributions for the initial and boost plans.

A comparison of representative dose distributions is shown in Figure 2. F‐3DCRT showed hot spots in the vicinity of the chest wall; whereas, I‐3DCRT gave the impression of a more uniform dose to the target. In VMAT, the dose in the target area was even more uniform, although the low‐dose area tended to expand. In contrast, bVMAT and hybrid tended to deliver a uniform dose to the target and simultaneously suppressed the low‐dose area in the lungs.

Table 2 compares the PTV dose indices and MU values with the OAR dose indices. Table 3 presents the *p*‐values for the different irradiation methods. The CI for VMAT and hybrid was >0.8. In particular, the VMAT technique showed better results for the PTV dose indices of D_98%_ and D_2%_. However, there were no significant differences among the different irradiation techniques with respect to D_mean_. Further, lung V_20Gy_ was lower with the VMAT technique, especially hybrid, which showed a significant reduction compared to F‐3DCRT (*p* < 0.05); MLD was also better with hybrid than with the other irradiation techniques (*p* < 0.05). In contrast, the VMAT and hybrid groups showed higher V_5Gy_ values. For the spinal cord, all techniques complied with the constraint of 50 Gy or less. Furthermore, no significant differences were observed between the irradiation techniques with regard to heart D_mean_ and esophageal D_mean_. The MU values tended to be higher for techniques that used VMAT, especially for the hybrid.

**TABLE 2:** Dosimetric parameters for each irradiation technique (mean ± standard deviation).

	Dose volume index	(a) F‐3DCRT	(b) I‐3DCRT	(c) VMAT	(d) bVMAT	(e) Hybrid
PTV	D_98%_ (Gy)	51.31 ± 2.24	51.17 ± 3.87	53.24 ± 3.65	53.48 ± 1.94	53.31 ± 2.99
	D_2%_ (Gy)	63.93 ± 2.53	62.65 ± 1.51	61.45 ± 0.31	62.88 ± 1.47	61.18 ± 0.63
	D_mean_ (%)	59.49 ± 0.51	59.22 ± 0.74	59.52 ± 0.28	59.65 ± 0.42	59.48 ± 0.73
	CI	0.34 ± 0.10	0.57 ± 0.10	0.86 ± 0.08	0.61 ± 0.12	0.83 ± 0.11
Lung	V_20Gy_ (%)	21.05 ± 10.56	20.86 ± 6.45	19.50 ± 7.38	19.98 ± 10.04	17.74 ± 7.86
	V_5Gy_ (%)	34.86 ± 14.48	39.82 ± 10.59	47.99 ± 17.42	36.95 ± 14.89	43.06 ± 15.44
	MLD (Gy)	11.34 ± 4.82	11.41 ± 3.36	11.24 ± 3.90	11.04 ± 4.77	9.56 ± 4.71
Cord	D_max_ (Gy)	42.85 ± 5.48	32.73 ± 6.73	32.64 ± 6.68	44.25 ± 8.17	34.47 ± 9.21
Heart	MHD (Gy)	7.68 ± 8.20	6.73 ± 8.93	6.68 ± 6.70	8.17 ± 7.46	9.21 ± 6.94
Esophagus	D_mean_ (Gy)	26.42 ± 12.94	29.18 ± 10.90	26.95 ± 11.53	26.55 ± 12.34	24.60 ± 10.95
Total MU (initial + boost)		527.39 ± 28.41	559.64 ± 23.04	860.66 ± 198.82	665.72 ± 97.11	1276.97 ± 246.77

Abbreviations: bVMAT, boost VMAT; CI, conformity index; D_max_, maximum dose; D_mean_, mean dose; D_XX%_, dose to XX% at the target volume; F‐3DCRT, forward planned‐3‐dimensional conformal radiotherapy; Hybrid, hybrid of fixed intensity‐modulated radiotherapy (IMRT) beams and VMAT beams; I‐3DCRT, inverse planned‐3‐dimensional conformal radiotherapy; MHD, mean heart dose; MLD, mean lung dose; MU, monitor unit; PTV, planning target volume; VMAT, volumetric modulated arc therapy.

**TABLE 3:** *p*‐values between each irradiation technique.

		*p*‐value									
		F‐3DCRT vs. I‐3DCRT	F‐3DCRT vs. VMAT	I‐3DCRT vs. VMAT	F‐3DCRT vs. bVMAT	I‐3DCRT vs. bVMAT	VMAT vs. bVMAT	F‐3DCRT vs. Hybrid	I‐3DCRT vs. Hybrid	VMAT vs. Hybrid	bVMAT vs. Hybrid
PTV	D_98%_ (Gy)	1.00	0.15	<0.01*	<0.01*	0.03*	0.74	0.16	0.13	0.74	0.89
	D_2%_ (Gy)	0.11	<0.01*	<0.01*	<0.01*	0.89	<0.01*	<0.01*	<0.01*	0.25	<0.01*
	D_mean_ (%)	0.72	0.51	0.33	0.03*	0.24	0.32	1.00	0.99	0.46	0.26
	CI	<0.01*	<0.01*	<0.01*	<0.01*	0.42	<0.01*	<0.01*	<0.01*	0.99	<0.01*
Lung	V_20Gy_ (%)	0.69	0.79	0.15	<0.01*	0.46	0.74	0.01*	0.05*	0.15	0.09
	V_5Gy_ (%)	0.02*	<0.01*	<0.01*	0.04*	0.06	<0.01*	<0.01*	0.41	0.11	0.01*
	MLD (Gy)	0.54	0.68	0.59	0.19	0.65	0.41	0.03*	0.02*	0.04*	0.13
Spinal cord	D_max_ (Gy)	<0.01*	<0.01*	0.79	0.34	<0.01*	<0.01*	<0.01*	0.46	0.38	<0.01*
Heart	MHD (Gy)	0.27	0.23	0.02*	0.04*	0.03*	0.64	0.09	0.01*	0.53	0.15
Esophagus	D_mean_ (Gy)	0.11	0.31	0.01*	0.91	0.15	0.64	0.22	0.01*	0.04*	0.11
Total MU (initial + boost)		<0.01*	<0.01*	<0.01*	<0.01*	<0.01*	<0.01*	<0.01*	<0.01*	<0.01*	<0.01*

Abbreviations: bVMAT, boost VMAT; CI, conformity index; D_max_, maxmum dose; D_mean_, mean dose; D_XX%_, dose to XX% at the target volume; F‐3DCRT, forward planned‐3‐dimensional conformal radiotherapy; Hybrid, hybrid of fixed intensity‐modulated radiotherapy (IMRT) beams and VMAT beams; I‐3DCRT, inverse planned‐3‐dimensional conformal radiotherapy; MHD, mean heart dose; MLD, mean lung dose; MU, monitor unit; PTV, planning target volume; VMAT, volumetric modulated arc therapy.

* *p*‐value <0.05.

As part of an additional experiment, we compared dose indices between the extension of the initial plan for all fractions and sequential plan changes across the three plans (I‐3DCRT, VMAT, and hybrid) on both the initial and boost CT images. Table 4 presents the results of each dosimetric parameters. Table 5 shows *p*‐values comparing initial plan extensions and sequential plan changes. For the heart, modifications in I‐3DCRT and VMAT led to a significant dose reduction (*p* < 0.05), while the hybrid resulted in a significant dose increase. For the lung dose index, there was no significant improvement observed between treatment strategies with or without plan changes. In addition, for CI, the initial plan extension on the boost CT image was poorer than on the initial CT image, especially in high‐precision irradiation methods like VMAT and hybrid. This indicates that the initial plan did not adapt to tumor changes.

**TABLE 4:** Dosimetric parameters for three irradiation techniques when the initial plan is extended for all fractions (mean ± standard deviation) and *p*‐values comparing initial plan extension to sequential plan changes.

		Initial plan extension on the initial CT images			Initial plan extension on the boost CT images		
		(b) I‐3DCRT	(c) VMAT	(e) Hybrid	(b) I‐3DCRT	(c) VMAT	(e) Hybrid
PTV	D_98%_ (Gy)	51.94 ± 2.17	55.70 ± 1.21	53.97 ± 3.12	50.26 ± 6.23	52.13 ± 5.15	50.26 ± 6.23
	D_2%_ (Gy)	63.05 ± 1.61	61.92 ± 0.30	62.12 ± 0.64	62.93 ± 1.26	58.11 ± 16.63	62.93 ± 1.26
	D_mean_ (%)	59.34 ± 0.74	59.76 ± 0.12	59.32 ± 0.91	59.52 ± 1.42	60.12 ± 0.8	59.52 ± 1.42
	CI	0.55 ± 0.12	0.85 ± 0.07	0.78 ± 0.13	0.61 ± 0.14	0.65 ± 0.12	0.61 ± 0.14
Lung	V_20Gy_ (%)	20.97 ± 6.29	19.05 ± 7.12	16.92 ± 7.50	18.41 ± 8.77	20.52 ± 8.41	18.41 ± 8.77
	V_5Gy_ (%)	38.82 ± 10.67	47.63 ± 17.60	40.72 ± 15.51	42.29 ± 16.42	48.95 ± 18.7	42.29 ± 16.42
	MLD (Gy)	11.50 ± 3.43	11.33 ± 3.93	10.35 ± 3.82	11.22 ± 4.62	12.18 ± 4.77	11.22 ± 4.62
Spinal cord	D_max_ (Gy)	34.30 ± 8.74	34.76 ± 8.35	39.17 ± 13.66	40.21 ± 12.17	36.78 ± 8.15	40.21 ± 12.17
Heart	MHD (Gy)	11.17 ± 10.71	8.63 ± 7.95	8.71 ± 8.39	7.39 ± 7.77	7.34 ± 7.35	7.39 ± 7.77
Esophagus	D_mean_ (Gy)	30.42 ± 10.39	28.06 ± 12.27	26.40 ± 11.92	25.89 ± 11.3	28.18 ± 12.54	25.89 ± 11.3

Abbreviations: bVMAT, boost VMAT; CI, conformity index; Dmax, maxmum dose; Dmean, mean dose; DXX%, dose to XX% at the target volume; F‐3DCRT, forward planned‐3‐dimensional conformal radiotherapy; Hybrid, hybrid of fixed intensity‐modulated radiotherapy (IMRT) beams and VMAT beams; I‐3DCRT, Inverse planned‐3‐dimensional conformal radiotherapy; MHD, mean heart dose; MLD, mean lung dose; MU, monitor unit; PTV, planning target volume; VMAT, volumetric modulated arc therapy.

**TABLE 5:** *p*‐values comparing irradiation techniques: Label A represents a sequential plan change, B denotes an initial plan extension on the initial CT image, and C indicates an initial plan extension on the boost CT image.

		A vs. B			A vs. C			B vs. C		
		(b) I‐3DCRT	(c) VMAT	(e) Hybrid	(b) I‐3DCRT	(b) I‐3DCRT	(b) I‐3DCRT	(b) I‐3DCRT	(b) I‐3DCRT	(b) I‐3DCRT
PTV	D_98%_ (Gy)	0.45	0.01*	0.24	0.95	0.64	0.15	0.46	0.01*	0.01*
	D_2%_ (Gy)	<0.01*	<0.01*	<0.01*	<0.01*	0.03	<0.01*	<0.01*	0.07	<0.01*
	D_mean_ (%)	0.81	<0.01*	0.06	0.13	<0.01*	0.69	0.08	0.19	0.55
	CI	0.33	0.55	0.05*	<0.01*	<0.01*	<0.01*	<0.01*	<0.01*	<0.01*
Lung	V_20Gy_ (%)	0.64	0.34	0.01*	0.02*	0.07	0.13	0.02*	0.02*	<0.01*
	V_5Gy_ (%)	0.13	0.27	<0.01*	0.17	0.22	0.41	0.03*	0.07	0.04*
	MLD (Gy)	0.38	0.74	0.59	0.02*	<0.01*	0.05*	<0.01*	0.01*	<0.01*
Spinal cord	D_max_ (Gy)	0.64	0.07	0.03*	0.01*	<0.01*	<0.01*	0.19	0.06	0.38
Heart	MHD (Gy)	0.04*	0.03*	0.03*	0.07	0.08	0.07	0.46	0.39	0.41
Esophagus	D_mean_ (Gy)	0.34	0.22	0.08	0.03*	0.02*	0.01*	0.99	0.89	0.69

Abbreviations: bVMAT, boost VMAT; CI, conformity index; Dmax, maxmum dose; Dmean, mean dose; DXX%, dose to XX% at the target volume; F‐3DCRT, forward planned‐3‐dimensional conformal radiotherapy; Hybrid, hybrid of fixed intensity‐modulated radiotherapy (IMRT) beams and VMAT beams; I‐3DCRT, inverse planned‐3dimensional conformal radiotherapy; MHD, mean heart dose; MLD, mean lung dose; MU, monitor unit; PTV, planning target volume; VMAT, volumetric modulated arc therapy.

* *p*‐value <0.05.

## DISCUSSION

Although treatment planning for LA‐NSCNC traditionally utilized 3DCRT, the effectiveness of techniques involving IMRT and VMAT has been demonstrated frequently.^11^, ^15^, ^16^, ^17^ IMRT adoption is increasing in Japan.^18^ However, not all facilities have access to IMRT, underscoring the continued relevance of 3DCRT in the modern age. In our study, we compared not only the basic 3DCRT and VMAT, but also analyzed 3DCRT with an inverse plan (I‐3DCRT) and scenarios where only a boost was used for VMAT. ART is effective against lung cancer from various perspectives.^19^ Notably, Harsolia et al. reported 39% and 44% reductions in PTV volumes with 4D‐offline ART and 4D‐online ART techniques, respectively; in turn leading to reduced mean lung doses by 26% and 31%, respectively.^20^ In our study, treatment planning employed different CT images for both the initial and boost plans, characterizing a limited offline ART approach. A notable tumor reduction was observed with a single re‐planning during the treatment course. However, no significant differences in lung volumes between the initial and boost CT images were detected in the current analysis. Likewise, there was no significant disparity in lung dose indices between treatment strategies with or without plan changes. This suggests that offline‐ART, incorporating just one re‐planning, provides limited reduction in lung dose throughout the treatment duration. However, lung cancer is often complicated by inflammation and atelectasis as well as deformation of the tumor itself, and many cases of lung cancer have significant differences in target location during the irradiation period. Therefore, it is essential to conduct more studies to determine the effectiveness of ART, especially since adjusting the plan could be instrumental in addressing changes in target positioning. To date, no studies have explored the evaluation of the five different treatment planning methods using different CT images, such as the two‐step method. Therefore, our study concentrated on these evaluations.

The CI for all techniques using VMAT was better than that obtained using 3DCRT. This is likely because VMAT irradiates from multiple directions, resulting in a higher dose uniformity than 3DCRT. The similarity in CI between hybrid and VMAT is in agreement with previous reports.^11^ Hybrid is effective in reducing lung dose because it incorporates fixed anteroposterior IMRT components and has a favorable impact on the target dose.

In context of the normal lung dose, V_20Gy_ showed a decreasing trend with the use of VMAT, whereas it was better with the use of hybrid (17.7 ± 7.9%, *n* = 13) in comparison with previous reports (23.3 ± 5.3%, *n* = 24),^11^ although it varied greatly from case to case. The V_5Gy_ increased significantly with the use of VMAT compared with 3DCRT, similar to that observed in previous reports.^21^ 3DCRT and hybrid showed a decreasing trend compared with VMAT because of the effective contribution of the fixed anteroposterior 3DCRT or IMRT components.

As for I‐3DCRT, it was possible to reduce the lung V_20Gy_ while improving the target CI compared with conventional F‐3DCRT. However, setting dose constraints, confirming the irradiation field, and determining other geometrical parameters are necessary for appropriate use. Regarding the change in treatment plan from F‐3DCRT to VMAT (i.e., bVMAT), a significant improvement in dose distribution (especially with respect to the target dose index) was confirmed compared to continuing with F‐3DCRT, and a reduction in the spinal cord dose was also possible, as well as a reduction in lung V_5Gy_. This suggests that if IMRT is not ready by the start of the initial planning, treatment may be initiated with 3DCRT. In addition, focusing on the heart, the MHD was similar between IMRT and 3DCRT in the present study, in agreement with the findings from previous studies.^8^

The hybrid technique demonstrated favorable dose characteristics; however, its high MU value is a notable concern. A shorter irradiation time is preferable when considering respiratory motion management such as breath‐hold, and a lower MU value is required to minimize low‐dose exposure.^22^ When implementing a treatment plan, the target dose constraints must be met; however, when using the irradiation method, the MU values must also be closely monitored.

This study had some limitations. First, this study was conducted at a single institution using a single treatment planner. Second, our study used a relatively small number of coplanar beams; therefore, studies on noncoplanar beams or increase in number of beams has not been conducted. Third, the optimal planning method may differ depending on the shape and location of the tumor. In this study, the number of cases was small, and each site was not examined separately. Therefore, further studies focusing on the tumor location are required. Fourth, the evaluation based on the boost CT might be subject to significant uncertainties related to lung volume changes and the deformable dose accumulation between the initial CT and the boost CT. Due to the limited number of cases in this study, a more detailed investigation of this influence is necessary.

In conclusion, this study compared the dosimetric characteristics of five different treatment planning techniques for LA‐NSCLC with sequential plan changes. The ART might be a useful tool for target shape changes, while offline‐ART with just one replanning might not provide sufficient reduction in lung dose throughout the treatment duration. The target CI was improved by using VMAT‐based techniques. In particular, the hybrid technique reduced the lung V_20Gy_ and V_5Gy_, but it should be noted that the MU value was higher. 3DCRT not only reduced exposure in the low‐dose region of the lungs but also may be used if IMRT is not ready by the start of initial planning.

## AUTHOR CONTRIBUTIONS

All authors had full access to the data in the study and take responsibility for the integrity of the data and the accuracy of the data analysis. Conceptualization, M.S and H.O.; Resources, M.S, T.K., K.M., S.A., T.A., M.M., N.S., H.S., U.K., H.N, and H.O.; Methodology, M.S.; Formal Analysis, M.S, and H.O.; Writing – Original Draft Preparation, M.S and H.O.; Writing – Review & Editing, M.S, T.K., K.M., S.A., T.A., M.M., N.S., H.S., U.K., H.N, and H.O.

## CONFLICT OF INTEREST STATEMENT

There is no conflict of interest with regard to this manuscript.
